# Breaking the ERK1/2 negative feedback loop: avoiding reactivation and acquisition of resistance by ERK1/2 to anti-cancer agents

**DOI:** 10.3389/fonc.2026.1948976

**Published:** 2026-09-28

**Authors:** Orel Slotki-Itzchakov, Sally Shpungin, Uri Nir

**Affiliations:** The Mina and Everard Goodman Faculty of Life-Sciences, Bar-Ilan University, Ramat-Gan, Israel

**Keywords:** cancer, ERK1/2, FER, MEK1/2, MTPN, signaling

## Abstract

The RAS-RAF-MEK-ERK signaling pathway plays a key regulatory role in the survival and proliferation of mammalian cells. Accordingly, deregulated activation of this cascade by a mutated K-RAS (mutK-RAS) is a major cause of cancer initiation and progression. These findings led to the development of synthetic inhibitors that target the mutK-RAS protein, as a therapeutic approach. However, the challenging design of synthetic compounds that effectively and selectively inhibit the mutK-RAS, and the high prevalence of acquired resistance to the mutK-RAS inhibitors, motivated also the development of synthetic inhibitors to downstream effectors of mutK-RAS, such as ERK1/2. Unfortunately, these putative anti-cancer drugs also exhibited limited efficacy, due to the reactivation of the inhibited ERK1/2 kinases by their deregulated upstream activators, RAF and MEK, an effect that led to an acquired resistance. Recently, several studies reported the targeting of novel, mutK-RAS-RAF-MEK-ERK sustaining proteins whose downregulation led to ERK1/2 inhibition, without subsequent ERK1/2 reactivation and without acquired resistance. The knockdown or inhibition of the intracellular tyrosine kinase FER, whose expression level is linked to poor prognosis of cancer patients, led to significant and persistent inhibition of ERK1/2, without subsequent reactivation. In an additional report, myotrophin (MTPN) was identified as a crucial mediator of ERK1/2 oncogenic activation. Accordingly, knockdown of MTPN blocked ERK1/2 activity without inducing later reactivation, or acquired resistance of these kinases. Here, we discuss these novel findings, which could pave the way for novel intervention approaches with deregulated ERK1/2 activation, with significant ramifications for the therapeutic targeting of the key K-RAS-ERK1/2 oncogenic pathway.

## 1 Introduction

The human RAS family contains three classic proto-oncogenes. Of these, *KRAS* is one of the most frequently mutated and deregulated oncogenes in human cancer (1). Making its encoded mutated product-mutK-RAS a key target for therapeutic intervention. Accordingly, drugs that inhibit mutK-RAS have been recently approved for the treatment of tumors driven by this oncogene (2). Though their clinical efficacy has been significantly improved, it is still limited, mainly due to treatment-associated resistance (3). This results in part from the fact that the RAS protein is prone to activating mutations, a feature that leads to acquired therapeutic resistance. One way to circumvent such therapeutic resistance of the mutK-RAS driven oncogenic cascade, is through targeting its downstream effectors.

Two major pathways are driven by K-RAS. One is the RAF-MEK1/2-ERK1/2 pathway (4), and the other one is the PI3K-AKT-mTOR cascade (5). It was recently found that the RAF-MEK-ERK, mitogen-activated protein kinase (MAPK) signaling, but not the PI3K-AKT-mTOR pathway, is sufficient to support mutK-RAS-dependent, pancreatic ductal-adenocarcinoma (PDAC) tumor growth. This underscores the key role of the RAF-MEK1/2- ERK1/2 cascade, as a central effector and mediator of mutK-RAS oncogenic activity in PDAC cells (6). Accordingly, efficacious targeting of these downstream effectors is expected to significantly impair the mutK-RAS-driven, malignant growth of PDAC tumors.

In this review, we focus on approaches and tools for targeting and modulating the RAF-MEK-ERK cascade, which plays pivotal roles in cell survival, proliferation, malignant transformation, and tumor progression (7, 8). Since RAF, similarly to RAS, can frequently acquire spontaneous activating mutations, that lead to therapeutic resistance (9), in the following sections we concentrate on describing the benefits and therapeutic challenges of MEK1/2, and ERK1/2 inhibitors when applied for cancer intervention and therapy.

## 2 Challenges in MEK1/2 and ERK1/2 inhibition for cancer intervention

MEK1/2 are phosphorylated and activated by RAF. They then further activate their downstream effectors, ERK1/2, by phosphorylating them on Thr202/185 and Tyr204/187 in ERK1 and 2, respectively (10). Thus, targeting MEK1/2 was suggested as a valuable intervention goal for inhibition of RAS-driven ERK1/2 activation, and for cancer therapy.

Most MEK inhibitors (MEKi), developed for the inactivation of MEK1/2, are non-ATP-competitors. Rather, they are allosteric inhibitors that lock MEK1/2 in a catalytically inactive state. Since they bind to domains with low homology to other protein kinases, they are highly selective and specific (11). Yet, clinical application of MEKi has failed to sustain long term efficacy due to resistance mechanisms imposed through upstream oncogenic mutations and rebound, resulting in RAF-MEK pathway reactivation (7, 12–14).

For example, the first MEK1/2 inhibitor, trametinib was approved for clinical applications, in 2013. However, this and other single agent therapies have not achieved long-term survival due to drug resistance linked to the reactivation of the RAF-MEK-ERK1/2 pathway. Furthermore, combined treatment with RAF and MEK1/2 inhibitors showed better response rates, but acquired resistance reflecting the reactivation of ERK1/2 remained a limiting factor in achieving sustained therapeutic efficacy (8).

The reactivation of ERK1/2 following inhibition could also explain the contradictory observation that although deregulation of the ERK signaling pathway is implicated in 40% of human cancers, and despite its therapeutic relevance, no ERK inhibitors have yet been approved by the FDA (**Table 1**). This reflects some basic challenges that must be addressed while developing an efficacious and reasonably safe ERK1/2 inhibitors for the clinic. Accordingly, despite extensive preclinical efforts and progress (**Table 1**), the development of selective ERK1/2 inhibitors continues to pose significant challenges primarily due to the high level of structural similarity across MAPKs, including p38 and JNK, and the frequent emergence of resistance that limits the duration of therapeutic efficacy (8). Hence, deciphering the mechanisms through which tumors acquire resistance to therapy, due to the reactivation of inhibited MEK1/2 and ERK1/2, is of a major importance, and continues to receive vast attention and focus.

**Table 1:** MEK1/2 and ERK1/2 inhibitors that are currently tested in various phases of clinical trials.

Inhibitor	Mechanism of inhibition	Clinical stage/status	Evidence of overcoming resistance
MEK1/2 inhibitors currently in clinical trails			
Trametinib (GSK1120212)	Allosteric, non-ATP-competitive MEK1/2 inhibitor	FDA approved (2013) in combination with dabrafenib for BRAF-mutant tumors; Phase III	Limited – resistance commonly occurs through MAPK pathway reactivation
Binimetinib (MEK162/ARRY-438162)	Allosteric MEK1/2 inhibitor	FDA/EMA approved in combination with encorafenib for BRAF(V600)-mutant melanoma; Phase III	Limited as monotherapy; combination strategies used to delay resistance
Cobimetinib (GDC0973)	Allosteric MEK1/2 inhibitor	FDA/EMA approved in combination with vemurafenib; Phase III	Limited- resistance can arise through MAPK reactivation
Selumetinib (AZD6244/ARRY-142886)	Allosteric MEK1/2 inhibitor	FDA approved for NF1-associated plexiform neurofibromas	Not primarily developed as a resistance-overcoming agent
Mirdametinib (PD-0325901)	Allosteric MEK1/2 inhibitor	FDA-approved for NF1-PN; combinations under investigation	Resistance remains a limitation; combination approaches investigated
CH5126766/VS-6766	Dual-mechanism MEK inhibitor: inhibits MEK catalytic activity and prevents MEK phosphorylation	Clinical development	Potentially stronger resistance suppression because it blocks both MEK activity and MEK activation
GDC0623	Allosteric MEK1/2 inhibitor	Clinical/preclinical development	Preclinical activity; resistance remains possible
ERK1/2 inhibitors currently in clinical trails			
Ulixertinib (BVD-523)	ATP-competitive ERK1/2 inhibitor	Phase I/II + combination with hydroxychloroquine is under investigation in a phase I clinical study *Ulixertinib (BVD-523) was granted an Expanded Access Program by the FDA.	Yes – it retains activity against several acquired resistance mechanisms, including mutations in KRAS, BRAF, and MEK.
Temuterkib (LY3214996)	ATP-competitive ERK1/2 inhibitor	Phase I	Yes- preclinical evidence in BRAF/MEK inhibitor-resistant models; clinical efficacy still limited
Rineterkib (LTT-462)	ATP-competitive ERK1/2 inhibitor	Phase I/II	Rineterkib retains potency in models resistant to standard treatments, including MEK inhibitors by effectively suppressing ERK-mediated bypass mechanisms.s
MK-8353 (SCH900353)	Dual-mechanism ERK inhibitor; ATP-competitive and prevent MEK-mediated phosphorylation of ERK1/2	Phase I	Yes, preclinical evidence against BRAF-inhibitor-resistant models
Ravoxertinib (GDC-0994)	ATP-competitive ERK1/2 inhibitor	Phase I	Ravoxertinib effectively suppresses tumor models with acquired resistance to upstream inhibitors, including BRAF inhibitors and MEK inhibitors, by targeting ERK at the terminal node of the MAPK cascade.
CC-90003	Covalent ERK1/2 inhibitor targeting a cysteine residue within the ATP- binding pocket.	Phase I; discontinued	CC-90003 effectively overcomes resistance to upstream RAF or MEK inhibitors by directly suppressing ERK signaling at the terminal node of the MAPK pathway.
Beroterkib (ASTX029)	Dual-mechanism ERK inhibitor; inhibits ERK catalytic activity and ERK phosphorylation	Phase I/II	Demonstrates activity in models resistant to RAF and MEK inhibitors *in vitro*.
Tizaterkib (AZD-0364)	ATP-competitive ERK1/2 inhibitor	Phase I/II	Preclinical data confirm that Tizaterkib overcomes resistance to RAF and MEK inhibitors by directly preventing ERK-mediated pathway reactivation. Tizaterkib
Edaxeterkib (KO-947)	ERK1/2 inhibitor with prolonged target residence	Phase I; discontinued	No convincing clinical evidence of overcoming resistance
ASN-007	ATP-competitive ERK1/2 inhibitor	Phase I; discontinued	Preclinical evidence against BRAF(V600E)-resistant tumors

The table summarizes the mechanisms of inhibition, clinical phases and putative acquired resistance, and evidence of mechanisms overcoming the reactivation and resistance of ERK1/2. Data is compiled from: Mao et al. *(*41*), and Schanbacher* et al. *(*2*),.*

## 3 Negative feedback regulation of ERK1/2 activation; implications to acquired therapeutic resistance

After activation by the upstream kinases C-RAF and MEK1/2, ERK1/2 are translocated to the cell nucleus (15). About 160 substrates of ERK1/2 have been discovered, many of which are transcription factors. Notably, among these substrates regulatory cytosolic proteins that restrain the activation of the RAS-RAF-MEK-ERK cascade were also identified. Aberrant ERK1/2 activation results in deregulated proliferation, survival, and malignant transformation of the affected cells. To avoid the adverse effects of deregulated ERK1/2, the activation process of ERK1/2 is tightly regulated and is controlled by several negative feedback pathways, some of which are driven by the activated ERK1/2 kinases, themselves. ERK1/2 can execute negative feedback loops through several trajectories (16). To downregulate C-RAF, cytoplasmic active ERK1/2 phosphorylate this upstream activating kinase on Ser29, Ser43, Ser289, Ser296, Ser301, and Ser642, which serve as RAF-inhibitory sites. These phosphorylation events impair the activation of C-RAF by interfering with its localization to the cell membrane, and its association with its upstream activator, RAS (17). Additionally, ERK1/2 phosphorylate MEK1 on Thr292, thereby inhibiting MEK1 activity and its ability to bind and activate ERK2 (18, 19). ERK1/2 also negatively regulate upstream activators of the of the RAS-RAF-MEK-ERK signaling cascade, namely growth factors, tyrosine kinase receptors (RTKs) and their cytoplasmic adaptors and scaffold docking effectors. This is exerted through ERK1/2 mediated phosphorylation of negative regulatory serine and threonine sites in receptors like EGFR, HER2, FGFR1, and IGFR1R, and their SOS1, and FRS2 alpha, effectors (20). ERK1/2 can also drive the induction of the dual specific phosphatases DUSP6 that directly dephosphorylates ERK1/2, thereby leading to its inactivation (21). Specifically, DUSP6 together with another dual specific phosphatase DUSP5, dephosphorylate ERK1 at Thr202/Tyr204, and ERK2 at Thr185 and Tyr187 (22), thereby inactivating them in the cytoplasm and nucleus, respectively (23, 24). The upregulation of DUSP6 by ERK1/2 is mediated through direct binding of the ERK1/2-responsive transcription factor ETS1 to the *DUSP6 gene* promoter (25, 26). Notably, ERK1/2 modulate the cellular level of DUSP6 not only at the transcriptional, but also at the post transcriptional level. ERK1/2 phosphorylate DUSP6 at positions Ser159/Ser197, thereby initiating ubiquitination of the protein and leading to its proteasomal degradation (27–29). Hence, while inhibition of ERK1/2 is expected to reduce the transcription rate of the *dusp6* gene, it will stabilize the DUSP6 protein and increase its cellular level.

Overall, activation of ERK1/2 leads to the onset of negative feedback inhibition of its upstream activators, RTKs, C-RAF and MEK1/2, and to the upregulation of its direct dual specific phosphatase, DUSP6. This, in turn, culminates in restricted and tightly regulated ERK1/2 activity. Importantly, inhibiting ERK1/2 is therefore expected to shut down these negative feedback inhibitory loops, thereby abrogating the intrinsic limitations and restrictions imposed on the ERK1/2 activation level. Consequently, this can enable the development of resistance to MEK1/2 and ERK1/2 inhibitors applied as potential anti-cancer agents. Collectively, the above findings imply that breaking the loops that reactivate ERK1/2 upon its inhibition, should be a major goal in the development of the next generation of efficacious, therapeutic ERK1/2 inhibitors.

## 4 Breaking the ERK1/2 negative feedback loop; avoiding reactivation and acquisition of resistance by ERK1/2 to anti-cancer agents

Inhibited ERK1/2 lose their ability to negatively regulate RAF and MEK1/2, thereby paving the way toward their reactivation. This is shown in **Figure 1** depicting the significant reactivation of ERK1/2 following their inhibition with their specific inhibitor - BVD-523 (Ulixertinib) (30). Interestingly, recent works showed that targeting the intracellular tyrosine kinase, FER, whose expression level is upregulated in numerous tumors and is linked to poor prognosis (31, 32), leads to inhibition of ERK1/2 with no observed reactivation process. In one study, an increased level of FER was detected in endometrial carcinoma (EC) tumors, when compared to the surrounding normal tissue. Knockdown of FER led to impaired proliferation, migration and invasion of EC cells and to an attenuated growth of EC xenografts in mice (33). It was further shown that the RAS-MAPK signaling cascade mediates the pro-oncogenic roles of FER in the studied EC cells. Specifically, knockdown of FER leads to a significant downregulation of the phosphorylated and activated- PERK1/2, in EC cells. Notably, the profound inhibition of ERK1/2 was persistent without any reactivation after 48 h of treatment. This could be explained by the unexpected downregulation of pan-RAS levels observed in FER deficient EC cells, an effect that would down-regulate the whole RAS-MAPK cascade and the activation level of ERK1/2. Accordingly, knockdown of FER also led to the downregulation of the phosphorylated MEK1/2 (PMEK1/2) kinases that are direct activators of ERK1/2. Thus, in EC cells FER seems to sustain the activity and functioning of the entire RAS-ERK1/2 pathway, and impairment of its regulatory role leads to a persistent suppression of ERK1/2, in the treated cancer cells. In another recent study, we investigated the role FER in sustaining the activation state of ERK1/2 in PDAC cells (34) that overexpress FER (32). Here again, knockdown of FER with siRNA, or treatment of the PDAC cells with E260, a selective, synthetic inhibitor of FER (35), led to the significant and persistent inhibition of ERK1/2, without any observed reactivation, even after extended exposure of the cells to E260, for 48 h. Consequently, these treatments arrested the growth and survival of the treated cells (33). In contrast, subjecting the PDAC cells to the specific ERK1/2 inhibitor BVD-523, led to a profound over-reactivation of these kinases (**Figure 1**). Importantly, cotreating the cells with both the ERK1/2 inhibitor, and the FER inhibitor, completely abolished the reactivation of the inhibited ERK1/2 kinases (**Figure 1**). It should be noted that in contrast to EC cells, in PDAC cells the inhibition of ERK1/2 upon the downregulation of FER, was not accompanied by the downregulation of the upstream ERK1/2 activators, MEK1/2 (34). This difference indicates that in PDAC cells, FER primarily sustains the active state of ERK1/2, rather than the activation state of their upstream activators. Several molecular events could underlie this unique effect. It was shown that FER can associate with ERK1/2 (36). Through this interaction, FER could shield ERK1/2 and modulate their susceptibility to their specific phosphatases DUSP5 and DUSP6. Knockdown of FER could therefore subject ERK1/2 to the constitutive dephosphorylating activity of DUSP6, thereby preventing its reactivation through a feedback-loop via its upstream activators RAF and MEK1/2. As noted above, a synthetic inhibitor of FER also led to a persistent inhibition of ERK1/2 without such an observed reactivation (34) (**Figure 1**). This could reflect the ability of the applied FER inhibitor, E260, to distort the tertiary structure of the FER kinase domain, thereby dissociating it from its cellular partners (35), including ERK1/2. An observation that could corroborate the involvement of phosphatase activity in the persistent inhibition of ERK1/2 following downregulation of FER in PDAC cells, stems from the fact that while exposure of ERK1/2 to their specific inhibitor BVD-523 leads to their profound reactivation, combining BVD-523 with E260, completely abolishes this reactivation (**Figure 1**). A similar observation of persistent inhibition of ERK1/2 was recently reported for the MTPN protein, which was found to mediate the endoplasmic -reticulum (ER) associated activation of ERK1/2, in colorectal cancer (CRC) cells. MTPN functions as a scaffold protein that mediates ERK binding to MEK, via a conserved ankyrin domain. Knockdown of MTPN persistently blocked ERK1/2 activation and signaling, without inducing subsequent ERK1/2 reactivation, thereby resulting in a significant attenuation of CRC xenograft growth in mice (37). This could reflect the requirement for MTPN in the MEK driven activation of ERK1/2, under the ER scope, in CRC cells. Alternatively, MTPN might not only mediate the activating association of MEK and ERK1/2, but it might also shield ERK1/2 from the suppressive, dephosphorylating activity of DUSP type phosphatases. Collectively, the above-described findings indicate that targeting some newly identified mediators of ERK1/2 activation in malignant cells can lead to a significant and persistent inhibition of ERK1/2 without ensuing reactivation. These novel observations can offer new avenues for the productive targeting of the oncogenic RAS-MAPK cascade, in cancer cells (**Figure 2**).

**Figure 1:**
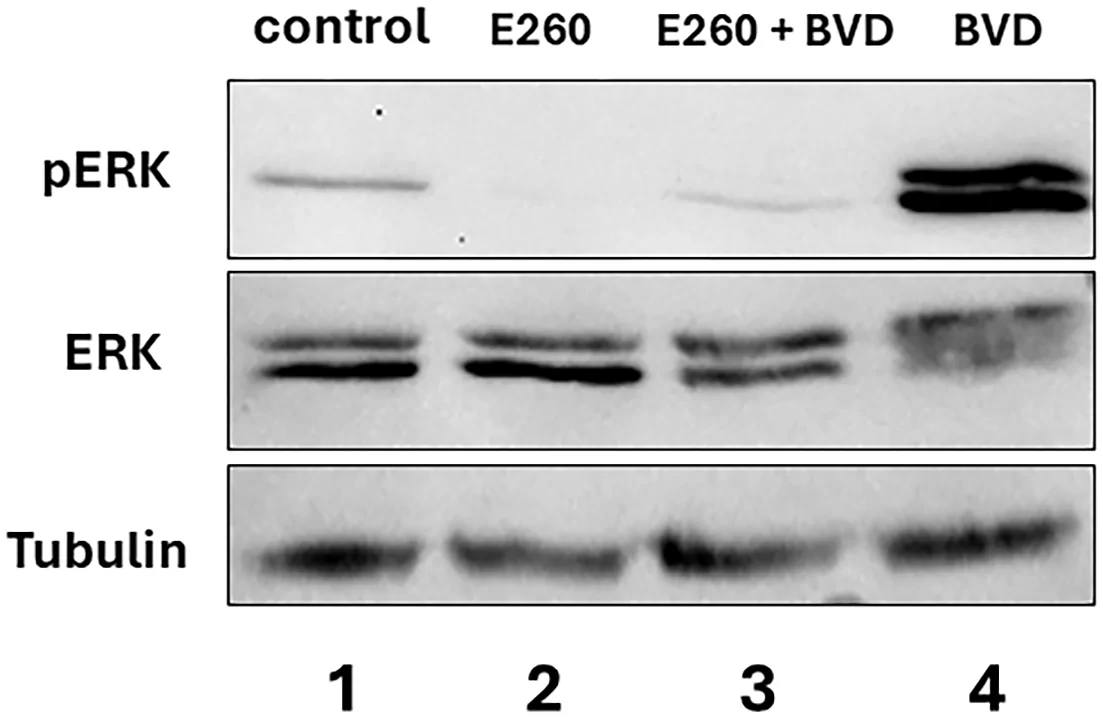
FER inhibitor prevents the reactivation of targeted ERK1/2 in PDAC cells. Metastatic SU.86.86 PDAC cells were left untreated (lane 1), treated for 48 hr with the FER inhibitor E260 (2.5 μM) alone (lane 2), with the FER inhibitor E260 and the ERK1/2 inhibitor BVD-523 (2.5 μM) together (lane 3), or with the ERK1/2 inhibitor BVD-523, alone (lane 4). The phosphorylation and activation states of ERK1/2 (pERK-in the upper panel) were determined in a western blot analysis, carried out with antibodies directed toward the phosphorylated Thr202/185, and Tyr204/187 aa residues in ERK1 and 2, respectively. The significant reactivation of ERK1/2 following their subjection to their genuine inhibitor -BVD-523 can be seen in lane 4 of the upper panel.

**Figure 2:**
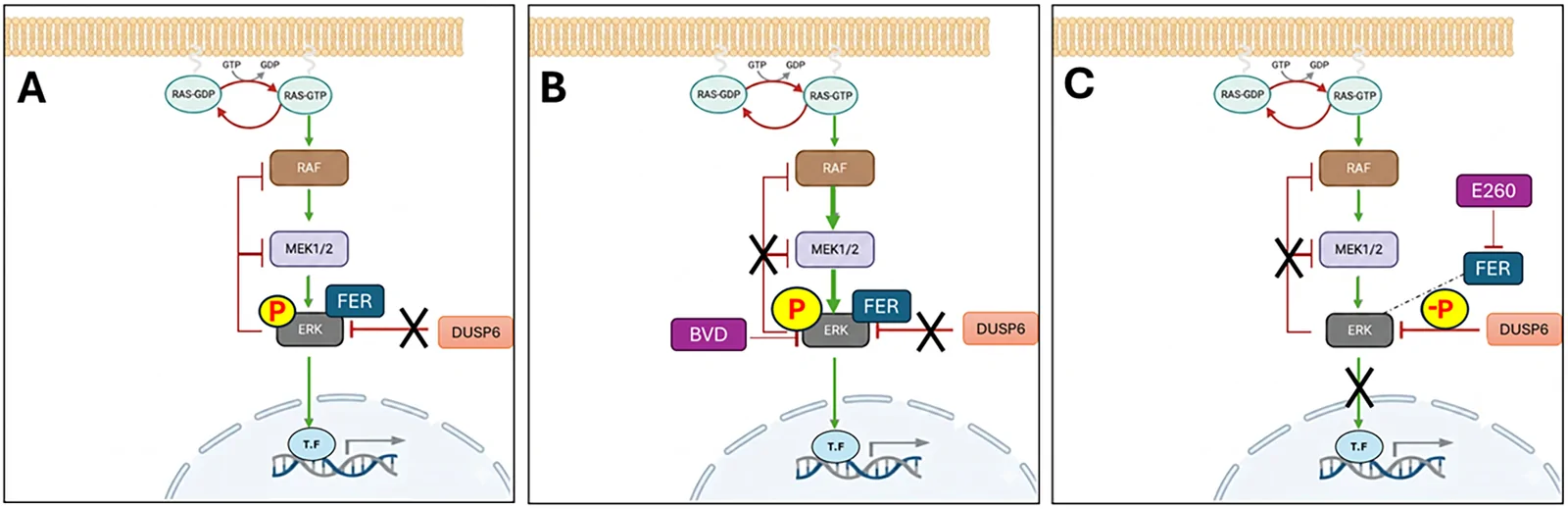
Breaking the ERK1/2 negative feedback loop- avoiding reactivation of targeted ERK1/2 in PDAC cells. **(A)** The signaling trajectory from activated RAS to ERK1/2. Activation state of ERK1/2 is sustained by FER and activated ERK1/2 exert inhibitory phosphorylation events on RAF and MEK1/2, thereby modulating its activation through inherent feedback loops. **(B)** Direct inhibition of ERK1/2 with a specific inhibitor like BVD-523, enables the signaling flow from RAS to ERK1/2 but shuts down the restrictive, negative feedback loops imposed by ERK1/2 on RAF and MEK. This leads to an intensive reactivation of ERK1/2. **(C)** Subjection of PDAC cells to the E260 inhibitor of FER, disables the FER directed maintenance of ERK1/2 activity thereby disabling the reactivation of ERK1/2.

## 5 Discussion

The main theme of this review is to describe novel modulators and regulators whose targeting can offer new approaches for persistently inhibiting ERK1/2 in malignant cells, while avoiding their feedback loop-driven reactivation by upstream activators. As we highlighted above, knockdown of FER or its downregulation with a selective synthetic inhibitor, leads to the persistent inhibition of ERK1/2 in EC and PDAC cells (33, 34). The finding that synthetic inhibitor of FER can exert persistent inhibition of ERK1/2 without subsequent reactivation, opens avenues for the development of new generation of synthetic compounds that could efficaciously interfere with the mutK-RAS-RAF-MEK-ERK oncogenic pathway. This goal can be achieved through the identification and targeting of newly discovered mediators of the ERK1/2 activation cascade. Since the oncogenic mutK-KRAS-MAPK pathway is a major and therapeutically challenging driver of the PDAC malignancy, the above-described findings open options for new strategies and approaches that will be applied for the treatment of this deadly cancer. However, it should be taken into consideration that inhibition of ERK1/2 not only unleashes the hyperactivation of RTKs and their downstream effectors RAS-RAF and MEK, but they might also induce and engage parallel survival and growth promoting pathway that includes PI3K-AKT-mTORC1, forming a central axis of resistance (38). Notably, we have shown before that downregulation of FER leads also to the deactivation of mTORC1 (33), thereby neutralizing also this circumventing trajectory that could foster resistance to ERK1/2 inhibitory anti-cancer agents.

Despite the promise of targeting FER, it should be noted that the regulatory engagement of FER in the oncogenic mutK-RAS-RAF-MEK1/2-ERK1/2 cascade, may vary among different cancer types. This notion is supported by the finding that in EC cells, FER sustains not only the activation state of ERK1/2, but also the cellular level of RAS and the activation state of MEK1/2 (33). Thus, optimal FER targeting tools and agents may vary for different cancers, and their development may therefore require distinct strategies. While considering this putative challenge, it should be noted that the therapeutic clinical application of a synthetic FER inhibitor like E260, can be prospected for several mutK-RAS driven cancers. This is inferred from the fact that E260 not only triggers the death of PDAC cells (33), but it also induces the death of mutK-RAS driven, metastatic colorectal cancer (CC) cells, and causes the regression of tumorigenic CC xenografts, elicited in immunocompromised mice (34). Significantly, the effects of E260 on these aggressive, mutK-RAS driven malignant cells and tumors is rather selective and was not seen in primary, epithelial and hematopoietic human cells (34). Accordingly, no adverse toxic effects were seen in mice subjected to daily injection of 50mg/Kg E260, for 25 days (34), Finally, E260 also exhibited favorable pharmacokinetic properties (34). Thus, there is a prospect of clinical application of a synthetic FER inhibitor, as an anti-cancer drug. However, while pursuing this path, its ability to prevent a long-term acquirement of drug resistance by ERK1/2, should also be verified. The potential diversity of newly discovered modulators of ERK1/2 activation is manifested by the recent identification of MTPN, a scaffold protein that mediates ERK1/2 binding to MEK1/2. This seems to drive the ER-associated activation of ERK1/2 in CRC cells. Notably, as was found for FER, knockdown of MTPN led to significant and persistent downregulation of ERK1/2, without the ensuing reactivation process of these kinases (37). This could reflect the requirement for MTPN in the MEK driven activation of ERK1/2. Alternatively, MTPN might not only mediate the activating association of MEK and ERK1/2, but it might also shield ERK1/2 from the suppressive dephosphorylating activity of DUSP type phosphatases. In either case, the newly opened possibilities of targeting ERK1/2 without subsequent reactivation, should provide new approaches for intervening with the oncogenic mutK-RAS-RAF-MEK1/2-ERK1/2 cascade (**Figure 2**), while reducing the potential of acquired therapeutic resistance. Furthermore, therapeutic targeting of new ERK1/2 modulators could be potentiated and enhanced by combining it with new, clinically approved mutK-RAS (39), RAF, or immune-checkpoint inhibitors (40).
